# Your brain is amazing: Let’s keep it that way

**DOI:** 10.1093/braincomms/fcac160

**Published:** 2022-07-22

**Authors:** Tara L Spires-Jones

**Affiliations:** Centre for Discovery Brain Sciences, UK Dementia Research Institute, University of Edinburgh, EH8 9JZ Edinburgh, UK

## Abstract

Our editor discusses brain resilience and how it can be harnessed to prevent diseases that cause dementia.

## Graphical Abstract

**Graphical Abstract:**
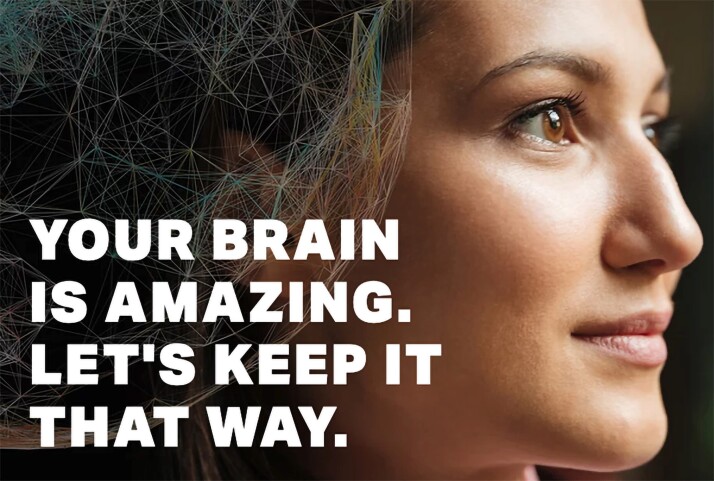

Welcome to Volume 4 Issue 4 of *Brain Communications*. This issue brings more fantastic papers in translational neuroscience from studies examining epilepsy patients^1^ to studies of induced pluripotent stem cell-derived motor neurons with mutations that cause motor neurone diseases.^2^ One topic that is close to my heart, namely brain resilience, is also highlighted in a *Brain Comms* paper. Kelley *et al.*^3^ examined brain tissue from 26 cognitively healthy participants in the Religious Orders Study and observed increased expression of synaptic genes in posterior cingulate cortex of Braak Stage III-IV participants compared with people with less tau pathology (Braak Stage I–II). In another study, Katsumi *et al.*^4^ investigated ‘super agers’, people whose memory function was on par with younger adults, and observed that they are less likely to experience post-operative delirium and that they have a larger anterior mid-cingulate cortex. Our group has also recently found an important role for synaptic resilience in maintaining healthy cognition during ageing.^5^

I find the study of resilience fascinating for many reasons. One is because I’ve been working to understand neurodegeneration for over 20 years, and it is more optimistic to instead study the other side of the coin, brain resilience. This field also has exceptional translational potential. In my view, the best news in the dementia field over the past century has come out of boosting brain resilience. Despite large increases in the prevalence of dementias due to an ageing population, the incidence of dementias in first world countries is *decreasing,*^6^ a fact attributed to better cardiovascular health and increasing education which are thought to build brain reserve. Since the best epidemiological estimates suggest at least one third of dementia cases could be prevented by modifying lifestyle risk factors,^7^ harnessing brain resilience has become an important public health issue. Here in Scotland, there is a new Brain Health Scotland initiative to promote brain health throughout people’s lives.^8^ The title of this editorial is the motto of Brain Health Scotland ‘Your Brain is Amazing. Let’s keep it that way.’ This initiative spans the life course starting with school children encouraging kids to keep their brains healthy by exercising, eating well, and avoiding injury. With the charity Alzheimer’s Scotland, the initiative also has brain health hubs, physical centres in communities with a poor track record of lifestyle risk factors for dementia where people can get advice on brain health and preventing dementia. I am hopeful that this initiative will further reduce dementia incidence in Scotland and will serve as a model for other countries to promote brain health. In future, I am also hopeful that research such as that by Kelley *et al.*^2^ and our group^5^ to understand the neurobiological underpinnings of brain resilience will allow development of therapeutics to prevent diseases that cause dementia for the two-thirds of people that cannot prevent them by leading a healthy lifestyle.

Well done, dear readers, for promoting your brain’s resilience by reading our journal!

The cover image for this issue comes from Michela Bassolino *et al.*^9^ and shows alterations in the perception of the affected limb in chronic patients with unilateral motor deficits after stroke.
